# The Map of Need: identifying and predicting the spatial distribution of financial hardship in Scotland’s veteran community

**DOI:** 10.1136/bmjmilitary-2020-001718

**Published:** 2021-03-04

**Authors:** Matthew D Kiernan, M Rodrigues, E Mann, P Stretesky, M A Defeyter

**Affiliations:** 1 Health and Life Sciences, Northumbria University, Newcastle upon Tyne, Tyne and Wear, UK; 2 Department of Social Sciences, Northumbria University, Newcastle upon Tyne, Tyne and Wear, UK

**Keywords:** occupational & industrial medicine, information management, public health

## Abstract

**Introduction:**

During military service, many household costs for both married and single service personnel are subsidised, and transition can leave veterans unprepared for the financial demands of civilian life. Armed Forces organisations such as Sailor, Soldier, Air Force Association (SSAFA) play a central role in understanding the financial challenges that UK veterans face and provide an insight into the financial hardship experienced by veterans. The aim of this study was to use SSAFA beneficiary data as a proxy to identify the nature of financial benefit, the spatial distribution of financial hardship in the Scottish SSAFA beneficiary community and explore factors that might predict where those recipients are located.

**Methods:**

Using an anonymised data set of Scottish SSAFA financial beneficiaries between 2014 and 2019, this study used a geographical methodology to identify the geospatial distribution of SSAFA benefit recipients and exploratory regression analysis to explore factors to explain where SSAFA beneficiaries are located.

**Results:**

Over half of benefit applicants (n=10 735) were concentrated in only 50 postcode districts, showing evidence of a clustered pattern, and modelling demonstrates association with area-level deprivation. The findings highlight strong association between older injured veterans and need for SSAFA beneficiary assistance.

**Conclusion:**

The findings demonstrate that beneficiaries were statistically clustered into areas of high deprivation, experiencing similar challenges to that of the wider population in these areas. Military service injury or disability was strongly associated with areas of high SSAFA benefit use and in those areas high unemployment was also a significant factor to consider.

**Key messages**

- Between 2014 and 2019, nearly a third of all Sailor, Soldier, Air Force Association (SSAFA) benefit was granted to provide accommodation or establish a safe living environment.
- Nearly a fifth of SSAFA benefit payments were granted to support daily subsistence.
- SSAFA beneficiaries were not homogeneously distributed but were in highly concentrated clusters.
- SSAFA beneficiaries were statistically clustered into areas of high deprivation, experiencing similar challenges to that of the wider population in these areas.
- There was a strong correlation between SSAFA beneficiaries and recipients of the War Pension Scheme.

## Introduction

During military service, many household costs for both married and single service personnel are subsidised and transition can leave veterans unprepared for the financial demands of civilian life.^1^ Moreover, some veterans face the challenge of entering employment and translating their experiences for civilian employers (Armed Forces Convenant, 2018). Once discharged from military service life, veterans can no longer access social networks for social support which adds further challenges for military veterans and their families.^2 3^

While there exists evidence on the challenges of transitioning to civilian life for UK Armed Forces veterans in terms of their mental and physical well-being, there is a paucity of research on veterans’ financial well-being. In the USA, the Department of Veteran Affairs supports the veteran population from end of service to end of life and there exists extensive research on the economic and demographic characteristics of ex-military personnel.^4^ US military veterans, in receipt of social security benefits, are less likely to be ‘poor’ or ‘near poor’ than the overall beneficiary population. Nevertheless, while support and benefits provided to US military veterans may mitigate against the risk of poverty and material hardship including housing, bill paying, medical bills and food insecurity, this financial support is eroded if the veteran is disabled.^5^ In addition, serious health conditions such as post-traumatic stress disorder and major depressive disorders appear to be associated with financial hardship in US Afghan and Iraq veterans.^6 7^ Conversely, there is little understanding of the financial challenges faced by UK Armed Forces veterans following transition to civilian life.

A recent report published by the Ministry of Defence (2018)^1^ highlights that as the circumstances of each devolved nation differ, the type and level of services should be tailored to local need. When considering the UK population as a whole, a significant challenge to exploring deprivation and financial hardship in any community is the lack of homogenisation of the Index of Multiple Deprivation (IMD) between each devolved nation. The IMD is the most comprehensive resource available to measure area-level deprivation in the UK, however, each specific IMD is not comparable due to different variables being used at different times.^8^ Therefore, to enable a comparison of the areas where the veteran population resides with that of the general population, this study focused on Scotland and the use of the Scottish Index of Multiple Deprivation (SIMD). There are 2.4 million UK Armed Forces veterans in Great Britain and approximately 10% (220 000) of veterans live in Scotland.^9^ Nevertheless, there exist little data on the location of veterans in Scotland seeking financial support from military charities.

In the absence of measures to determine the level of need and location of veterans seeking beneficiary support from military charities, there is a need to explore a proxy measure. While there has been extensive research regarding area-level hardship in the wider population within the UK,^8 10–12^ there is little, if any, research which has been undertaken to determine area-level hardship in the veteran population and whether the socioeconomic and geographical characteristics of that hardship differ from that of the general population. Moreover, within the UK there is no single government department that maintains a constant record of veterans: the health and social needs of UK veterans are provided for by the NHS and local government social services, alongside the wider population. When undertaking research on the UK veteran population, the persistent challenge is always the lack of a current record on all who have served and their current whereabouts.

In the UK, Armed Forces charities provide important support to the veteran community following military service. The oldest UK Armed Forces charity, Sailor, Soldier, Air Force Association (SSAFA), plays a vital role in supporting military personnel facing financial hardship and in 2018 supported 82 000 people with charitable services.^13^ SSAFA provides means tested assistance to anyone who is currently serving or has ever served in the British Armed Forces and their families. All applications for financial assistance from SSAFA are considered against a rigorous financial criterion. Organisations such as SSAFA play a central role in understanding the financial challenges that UK veterans face. This study sits within a wider UK project, known as the Map of Need (MoN). The MoN provides an important Public Observatory function on the veterans’ community, enabling informed funding decisions based on evidence and data. More specifically, the MoN uses current service usage, or proxy data from the state and the third sector to determine future service need at the regional, local government and postcode levels across the UK. The MoN team have established legal data sharing agreements with 14 state and charity sector organisations, such as SSAFA, who share anonymised usage data on an annual basis. By using proxy data from organisations such as SSAFA it is possible to gain an insight into the financial hardship experienced by veterans and the geospatial footprint of that population, and explore the factors that predict who will seek benefit and where they are located.

The aim of this study was to use SSAFA beneficiary data as a proxy to identify the nature of financial benefit and the spatial distribution of financial hardship in the Scottish SSAFA beneficiary community, and explore factors that might predict where those recipients are located.

## Method

Using an anonymised data set of all Scottish SSAFA financial beneficiaries between 2014 and 2019, this study used a geographical methodology to identify the distribution of SSAFA benefit recipients. In addition, exploratory regression analysis was used to explore factors that could help to explain where SSAFA beneficiaries are located.

This study measured SSAFA benefit cases through a yearly average count and a yearly average crude rate. Crude rates were calculated with the available population figures from the 2011 UK census, calculating the total number of SSAFA cases per 10 000 population. The measure of yearly average case prevalence was calculated by dividing the crude rate in an area by six, the number of years in the data time frame. In this study, demand and prevalence were combined with a unity-based normalisation to give a single summary measure to estimate SSAFA benefit need.

To determine the statistical relevance of the geographical hot spots (p<0.05), the Getis-Ord Gi* spatial statistic was used,^14^ testing if the spatial pattern could not have happened due to random chance. The 2020 SIMD was then used to map the conditions of the communities in which veterans live.

Exploratory regression analysis was used to explore factors associated with SSAFA cases in postcode districts. Regarding the sample, postcode counts were aggregated at the postcode district and all the postcode districts with less than five cases were excluded from the analysis. The dependant variable was the yearly average number of SSAFA cases (2014–2019). All the predictor variables were normalised to a common decimal scale (0–1), where 1 would represent the postcode with the highest value. Because of the way that deprivation indexes rank areas, the SIMD was inversely normalised so that the postcode district with the lowest mean rank would have a normalised value of 1 (most deprived) and the postcode with the highest mean rank should have a normalised value of 0 (less deprived). This normalisation was undertaken so that any increase in the SIMD deprivation scale increased the overall deprivation score in the postcodes.

Table 1 shows the data sets used in the initial exploratory regression. All the variables were available at the postcode district level and selected due to their availability and potential to influence the need to seek SSAFA benefit. Exploratory regression with ordinary least squares (OLS) modelling was used to determine those variables that would be included in the final set of explanatory variables. The criterion to pass this initial exploratory analysis was set at a minimum acceptable cut-off value of p=0.5.

**Table 1:** Independent variables used in the initial exploratory regression model

Independent variable	Indicator type	Description
AFCS Recipients—veterans	Count	AFCS: It provides compensation for all injuries, ill-health and death attributable to Service where the cause occurred on or after 6 April 2005.*
Armed Forces Pension Schemes (AFPS) Recipients—veterans	Count	AFPS: Veterans in receipt of their pension under AFPS 75 and AFPS 05. AFPS 75—introduced in 1975 and closed to new members from 6 April 2005. Pension benefits are based on rank and time served. AFPS 05—introduced on 6 April 2005. Pension benefits are based on time served and final salary.*
WPS Recipients—veterans	Count	WPS: It provides compensation for all injuries, ill-health and death attributable to Service where the cause occurred until 5 April 2005.*
Property price	Pounds	Average price of residential properties sold in 2019.†
SIMD	Rank	SIMD for the year of 2020.‡
Income deprived	Count	Number of people who are income deprived. Source: SIMD2020.‡
Unemployment	Count	Number of people who are employment deprived. Source: SIMD2020.‡
Illness factor	Standardised ratio	Comparative illness factor: standardised ratio. Source: SIMD2020.‡
Alcohol	Standardised ratio	Hospital stays related to alcohol misuse: standardised ratio. Source: SIMD2020.‡
Depression	Percentage	Proportion of population being prescribed drugs for anxiety, depression or psychosis. Source: SIMD2020.‡
No qualifications	Standardised ratio	Working age people with no qualifications: standardised ratio. Source: SIMD2020.‡
Drive General Practioner (GP) / Family Doctor	Time (minutes)	Average drive time to a GP surgery in minutes. Source: SIMD2020.‡
Public transport GP	Time (minutes)	Public transport travel time to a GP surgery in minutes. Source: SIMD2020.‡
Crime	Count	Number of recorded crimes of violence, sexual offences, domestic housebreaking, vandalism, drugs offences and common assault. Source: SIMD2020.‡
No central heating	Count	Number of people in households without central heating. Source: SIMD2020.‡

*Location of armed forces pension and compensation recipients: 2019 https://www.gov.uk/government/statistics/location-of-armed-forces-pension-and-compensation-recipients-2019.

†Average price by postcode: https://housepricescotland.com/.

‡SIMD: https://simd.scot/.

AFCS, Armed Forces Compensation Scheme; SIMD, Scottish Index of Multiple Deprivation; WPS, War Pension Scheme.

## Results

Between 2014 and 2019, 286 postcodes districts across Scotland had at least five SSAFA beneficiaries. Table 2 outlines what benefit was granted for and shows that the most significant assistance given was to provide accommodation or establish a safe living environment (29.34%, n=6087), with just under a fifth of benefit payments being granted to support daily subsistence (18.92%, n=3926) which includes buying food. The 286 postcode districts identified a total of 20 747 SSAFA benefit recipients, an average concentration of 73 recipients per postcode district or a yearly average of 12 recipients per postcode district. However, when examining the spatial distribution of the beneficiaries it was identified that the recipients were not homogeneously distributed but were in highly concentrated clusters. Of the 286 identified, 52% (n=10 735) of recipients were concentrated in only 50 postcode districts showing evidence of a clustered pattern.

**Table 2:** Purpose of assistance (2014/2019)

Assistance	Count	Percentage
Housing: providing accommodation and setting up home (white goods/brown goods)	6087	29.34%
Subsistence for daily living (including food)	3926	18.92%
Payment to partner organisation to aid beneficiary	3628	17.49%
Debt	2443	11.78%
Housing assistance (rent, repair, etc)	1443	6.96%
Mobility assistance and home adaptation	1157	5.58%
Non-specified assistance	720	3.47%
Re-training/education	671	3.23%
Funeral costs	311	1.50%
Care costs	269	1.30%
Respite breaks	73	0.35%
Support for retired commonwealth service personnel	19	0.09%
Total	20 747	100%

Between 2014 and 2019 the mean of SSAFA benefit recipients was 3458 (SD=831) beneficiaries per year. Among the postcodes that had at least five SSAFA beneficiaries between 2014 and 2019, 62% (n=12 907) were residing in Scotland’s central belt. The highest prevalence rates were found in North Ayrshire (yearly average of 18 beneficiaries per 10 000 population) and in the East and South Ayrshire (yearly average of 16 beneficiaries per 10 000 population).

**Figure 1:**
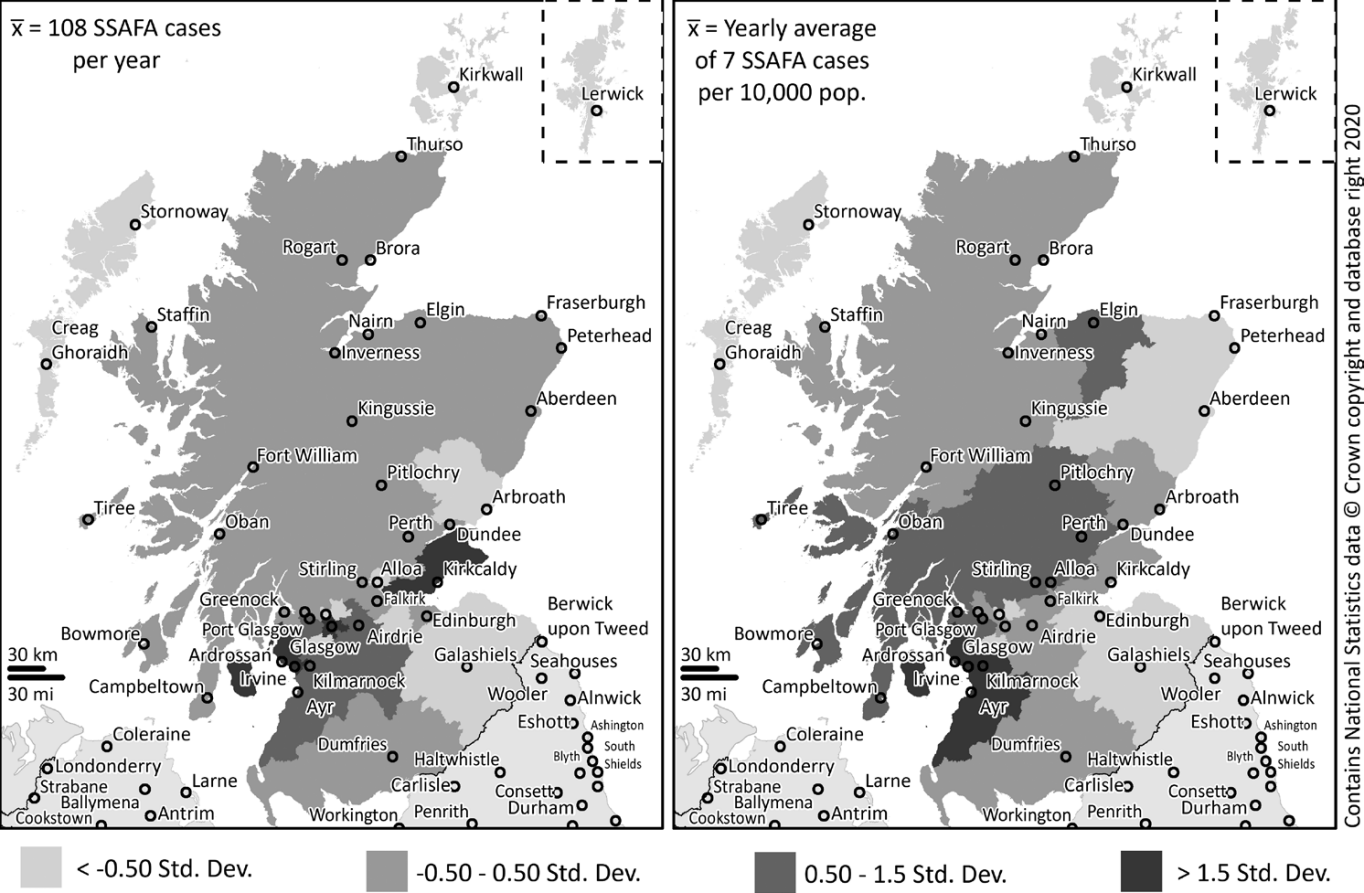
Sailor, Soldier, Air Force Association (SSAFA) welfare demand (on the left) and prevalence (on the right) across Scotland’s local authorities (2014/2019).

Figure 1 shows SSAFA beneficiaries and prevalence across Scotland’s local authorities, the dark grey areas indicating where benefit demand (on the left) and benefit prevalence (on the right) were greater than 1.5 SD above the respective yearly mean. The lighter grey areas are where demand and prevalence were less than 0.5 SD below the yearly mean. The classification of −0.50 to 0.50 indicates the areas where demand and prevalence were approximately the same as the yearly average (mean) across Scotland’s local authorities.

**Figure 2:**
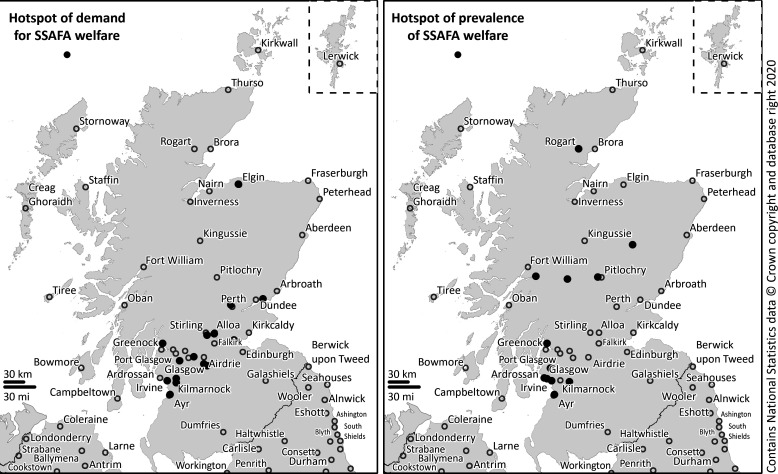
Significant (p<0.05) hot spots of Sailor, Soldier, Air Force Association (SSAFA) welfare demand and prevalence (2014/2019).

Figure 2 demonstrates that calculating hot spots from the spatial autocorrelation analysis of the distribution of SSAFA beneficiaries would mean that the sum of cases within each postcode would be the only variable used to determine a hot spot (Figure 2, on the left). Alternatively, to calculate the hot spots of welfare prevalence (Figure 2, on the right) would miss all the postcodes with a high demand among an equally high background population.

**Figure 3:**
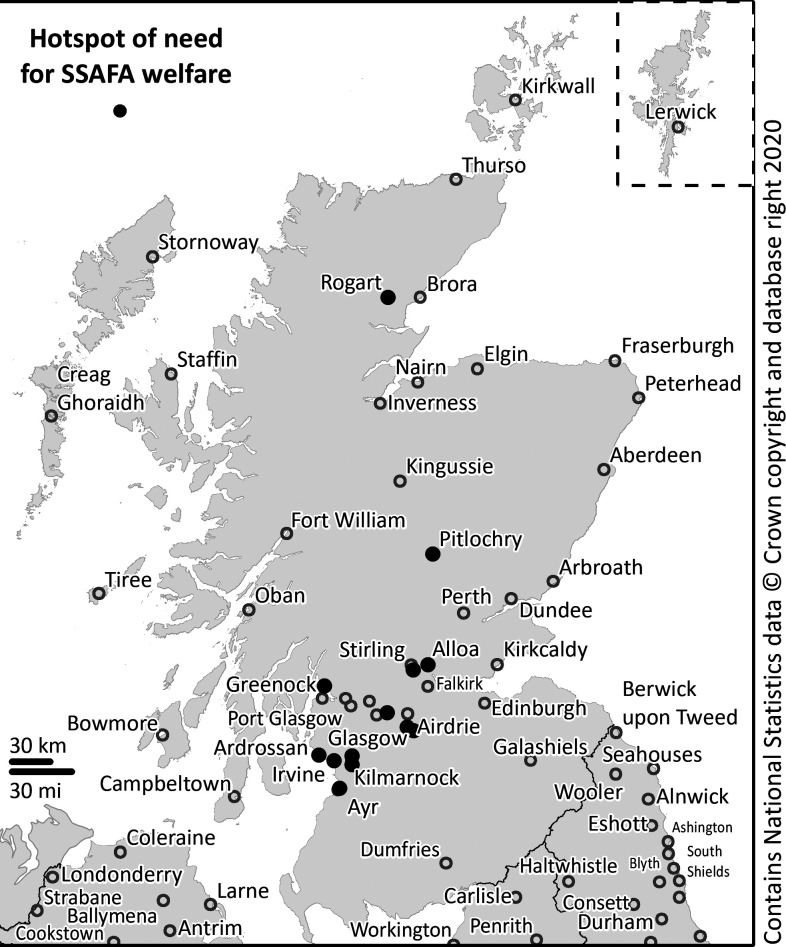
Significant (p<0.05) hot spots of need for Sailor, Soldier, Air Force Association (SSAFA) welfare (2014/2019).

Figure 3 shows where the demand is, while also considering the prevalence among the background population. To calculate the hot spots in Figure 3, demand and prevalence were combined with a unity-based normalisation. When examining the spatial distribution of the hot spots (Figure 3) it again shows that beneficiary need is not evenly distributed, but disproportionately clustered in a few postcodes. Across Scotland it was possible to find 13 statistically significant postcodes concentrating 19% (n=3874) of the beneficiaries.

Table 3 shows the eight variables which passed minimum acceptable cut-off value of p=0.5, and were included in the final regression models.

**Table 3:** Initial exploratory regression showing only the variables which passed the cut-off p value

Independent variable	Ordinary least squares estimation number of observations: 286 R-squared: 0.532 Akaike info: 2105.81 Jarque-Bera prob: 0.000			Summary of variable significance among all possible variable combinations		
	Coefficient	t-statistic	P value	% Significant	% Negative	% Positive
WPS recipients—Veterans	20.374	2.312	0.022	73.05%	0%	100%
No central heating	−28.46	−2.931	0.004	70.72%	80.88%	19.12%
SIMD	14.809	1.605	0.11	18.41%	6.13%	93.87%
AFPS recipients—Veterans	11.502	1.117	0.265	50%	0.67%	99.33%
AFCS recipients—Veterans	6.749	1.111	0.267	25%	6.25%	93.75%
No qualifications	−10.835	−0.998	0.319	7.70%	78.56%	21.44%
Unemployment	28.187	0.844	0.399	49.41%	16.24%	83.76%
Alcohol	−6.504	−0.802	0.424	7.35%	81.20%	18.80%

R-squared: indicates how much variation of a dependent variable is explained by the independent variable(s).

Akaike info: estimates the relative amount of information lost by a given model: the less information a model loses, the higher the quality of that model.

Jarque-Bera prob: indicates if the data have a normal distribution. If it is far from zero, it signals the data do not have a normal distribution.

Coefficient: indicates the change in the dependent variable for one unit of change in the independent variable. A negative coefficient suggests that as the independent variable increases, the dependent variable tends to decrease.

t-statistic: the coefficient divided by its standard error, thus estimating the likelihood that the regression coefficient is different from zero.

p value: estimates what the odds are of the results to have happened. The lower it is, the less likely the results could have happened due to random chance.

AFCS, Armed Forces Compensation Scheme; SIMD, Scottish Index of Multiple Deprivation; WPS, War Pension Scheme.

The final variables selected were used to integrate three regression models, an OLS estimation and two spatial models integrating distance weights (Table 4). Weights were calculated with a bandwidth of 162 km, the minimum value for which there was one neighbour for each postcode district. A spatial lag model integrates a variable that averages the neighbouring values of a location. This reflects the spatial dependence in the data, measuring the average influence on observations by their neighbouring observations. This method aims to account for spatial autocorrelation in the model, with the weight for each observation being dependent on its neighbours through a weight’s matrix.

**Table 4:** Summary of regression output for the selected eight best predictors

Independent variable	Ordinary least squares estimation			Spatial* lag model—Maximum likelihood estimation			Spatial* error model—Maximum likelihood estimation		
	R-squared: 0.529 Akaike info: 2093.5 Jarque-Bera prob: 0.000			R-squared: 0.541 Akaike info: 2089.7 Breusch-Pagan test: 0.000			R-squared: 0.537 Akaike info: 2090.43 Breusch-Pagan test: 0.000		
	Coeff.	t-stat.	P value	Coeff.	z-value	P value	Coeff.	z-value	P value
No central heating	−32.188	−3.827	0.000	−30.778	−3.749	0.000	−31.776	−3.875	0.000
Unemployment	39.025	5.561	0.000	37.957	5.526	0.000	39.568	5.71	0.000
WPS recipients – Veterans	21.492	2.594	0.01	18.506	2.283	0.022	18.31	2.236	0.025
SIMD	13.248	1.911	0.057	13.136	1.947	0.052	12.984	1.872	0.061
AFCS recipients – Veterans	7.389	1.242	0.215	6.794	1.172	0.241	6.99	1.208	0.227
No qualifications	−8.976	−1.095	0.275	−9.342	−1.171	0.242	−9.837	−1.207	0.227
AFPS recipients – Veterans	10.607	1.08	0.281	15.126	1.564	0.118	14.674	1.5	0.134
Alcohol	−7.212	−1.05	0.295	−7.134	−1.067	0.286	−8.736	−1.27	0.204
Spatial lag	---	---	---	0.372	2.672	0.008	---	---	---
Lambda	---	---	---	---	---	---	0.450	2.224	0.026

Breusch-Pagan: tests whether the variance of the spatial errors from a regression is dependent on the values of the independent variables.

Spatial lag and the spatially correlated errors (λ) reflect the spatial dependence inherent, measuring the average influence on observations by their neighbouring observations. Both coefficients have a positive effect and are highly significant. As a result, the general model fit improved. The effects of other independent variables remain virtually the same.

*Spatial distance weights: (a) Bandwidth: 162 km; (b) Min neighbours: 1; (c) Max neighbours: 203; (d) Mean neighbours: 130.

AFCS, Armed Forces Compensation Scheme; SIMD, Scottish Index of Multiple Deprivation; WPS, War Pension Scheme.

Overall, the models show that the variables regarding the presence of War Pension Scheme (WPS) recipients, absence of central heating and unemployment were highly significant. However, the absence of central heating had a negative relationship, meaning that postcodes with a high number of people in households without central heating would equate to lower number of SSAFA recipients. The presence of WPS recipients and unemployment had a positive relationship in the distribution of SSAFA beneficiaries. What this suggests is that, at the postcode district level, an increase in the number of WPS recipients and people who are employment deprived was coupled with an increase in the number of SSAFA beneficiaries.

Regarding SIMD, it almost passed the significance level (p<0.05), while maintaining a positive relationship. This suggests that in Scotland an increase in area-level deprivation has correspondence with an increase in the number of SSAFA recipients. The remaining predictors did not perform as well, and none was even close to being statistically significant in explaining the distribution of SSAFA recipients.

Overall, the models demonstrate that where the beneficiaries were located had a good correlation with where you would expect to find benefit recipients in the wider population, that is, deprived areas with high unemployment figures.

The WPS is a pension awarded for military service injury or disability prior to 2005 and pre-dates the current Armed Forces Compensation Scheme. Therefore, WPS is generally associated with older veterans with service-related disabilities. The strong association with the presence of WPS suggests a strong association between older injured veterans and the need for SSAFA beneficiary assistance.

The spatial lag and spatial error models improved the model fit as shown with the increase in regression fit and lower Akaike information criterion (Table 4). However, despite being able to explain about 50% of the variation in SSAFA beneficiaries, the results suggest a biased model. The Breusch-Pagan test indicated that the relationships between the explanatory variables and SSAFA beneficiaries was non-stationary, meaning that although the model performed well in one area, it performed poorly in others.

This means that although the model detected strong predictors for the presence of SSAFA beneficiaries across Scotland (eg, WPS recipients and unemployment), the same variables could be strong predictors in some areas, but weak predictors in others. Therefore, it is concluded that there were explanatory variables missing, due to the non-constant variance that the models produced, that could accurately predict the location of SSAFA beneficiaries in Scotland. Although the proxy data cannot reliably predict the location of SSAFA beneficiaries, they do suggest a possible association between older veterans with service-related injury or disability, unemployment and financial hardship (the need for SSAFA benefit).

## Discussion

The findings of this study have shown that the most significant need for SSAFA financial benefit in Scotland was for accommodation provision or to establish a safe living environment. The need of financial assistance for housing is not unique to the veterans’ community and is widely observed in the general population across both Scotland and the wider UK. For instance, Dowler and Lambie-Mumford^15^ report that fuel, housing and food costs are having severe consequences on many UK households. It is evident that poor quality housing and poor housing conditions are associated with poor physical and mental health including respiratory conditions, cardiovascular diseases, stress, depression and anxiety.^16^ When considering food cost, the second most significant need for SSAFA benefit was to support daily subsistence, which includes assistance for buying food. Food insecurity is ‘a condition that occurs when individuals and households do not have regular access to a supply of healthy and nutritious food to meet their dietary needs. This has become a substantial problem in the advanced capitalist world, with sizeable portions of affluent countries struggling to eat healthily every day’.^17 18^ In the UK, households in the lowest income decile do not have sufficient income to achieve a healthy diet as directed by the NHS Eatwell Guide.^19^ A key barrier for low-income households to achieve a healthy diet is the cost of healthy food items such as fruit and vegetables which are more expensive than high energy dense food with poor nutritional value.^20 21^ Furthermore, food outlets selling less healthy foods including fast-food outlets are more likely to be concentrated in disadvantaged neighbourhoods and this further contributes to the rising health inequalities.^19^ Unsurprisingly, adults from low-income households are more likely to become obese or suffer from diet-related ill-health such as cardiovascular disease, type 2 diabetes and osteoporosis.^16 19^ Research in the USA on veterans and food insecurity identified that while veterans are less likely to report hunger or seek nutritional assistance than non-veterans, food-insecure veterans were more likely to be younger, suffer from mental health conditions, and report difficulty with physical function and mobility.^22 23^

When considering the distribution of veterans receiving SSAFA benefit, they are not homogeneously distributed among the population of Scotland. Over 50% are clustered into 50 postcode districts out of a total of 286 that had at least five SSAFA beneficiaries and just under a fifth are clustered into 13 statistically significant postcodes in Ayrshire and Glasgow. Most notably, the statistical modelling demonstrated a good correlation between SSAFA beneficiaries and where you would expect to find benefit recipients in the wider population. Glasgow contains the largest share of deprived areas of any town or city in Scotland with over a third of Glasgow’s residents living in areas which fall within the 10% most deprived neighbourhoods in Scotland.^24^ Most significantly, this shows that Scottish SSAFA beneficiaries are located in areas of higher deprivation with higher levels of unemployment. Therefore, it can be argued that the difficulties that they are experiencing are not because they are veterans, but because they are living in areas where the wider population face the same challenges. The spatial clustering of deprivation can have wide-reaching impacts on all individuals living in a deprived community.^25^ Moreover, Sampson *et al* 26 discovered that health and well-being are impacted by the community in which one resides, and by the conditions of surrounding communities. This community effects research supports the position that SSAFA beneficiaries living in high deprivation areas are likely confronted with a social-structural milieu that may intensify any economic hardship they face by limiting access to beneficial community resources and helpful informal networks.^27^ We recommend that future research examine the pathways and extent of the clustering of deprivation on veterans facing economic hardship.

These findings are markedly different to those of the veteran population in England. A similar study undertaken on the veteran population in England^28^ found that similar to Scotland, the population is not homogenously distributed, but clustered. However, unlike Scottish beneficiaries, who are clustered into areas of high deprivation, SSAFA beneficiaries in England were located in areas of low deprivation but are found to still experience the same challenges as their Scottish counterparts (housing and food security).

While the models performed poorly in predicting why SSAFA beneficiaries lived where they did, it demonstrated a strong correlation between beneficiaries and recipients of WPS. In areas where high number of SSAFA beneficiaries are found there are also high numbers of WPS beneficiaries. The WPS is a financial award or pension given to retired service personnel who have a military service attributable injury or illness that predates 2005.

The study aimed to highlight the main areas of concentration of SSAFA benefit recipients in Scotland and determine the relative influence of a set of publicly available predictor variables to the location of those recipients. However, the data do not consider the awareness and availability of help. For example, the spatial distribution may only show those veterans seeking help which may result from a greater awareness and availability of help in some areas more than others. Additionally, the availability of public data that could reliably serve as a predictor was limited and the pitfalls of using an incomplete set of predictors are acknowledged.

Finally, other limitations are related to the broad scope of assistance provided. SSAFA assistance ranges from cases that might be facing temporary hardship to complex and long-term hardship. This study considered all the cases despite their purpose of assistance due to the impossibility of defining a poverty threshold. Therefore, it should be noted that some categories of assistance could have a better fit to the tested models, which is something that needs to be taken in consideration for future research.

Despite these limitations, the findings demonstrate that beneficiaries were statistically clustered into areas of high deprivation, experiencing similar challenges to that of the wider population in these areas. Military service injury or disability was strongly associated with areas of high SSAFA benefit use and in those areas high unemployment was also a significant factor to consider.

## Data Availability

Data are available upon reasonable request. Data cannot be shared publicly because the data belong to a third party, SSAFA, and are shared under a legal data sharing agreement for the purpose of this study. In addition, the data contain sensitive information on the location of vulnerable veterans. Data from the study are available upon request from MDK, Faculty of Health and Life Sciences, Northumbria University, for researchers who meet the criteria for access to confidential data and upon agreement of SSAFA.
